# Free breathing whole-heart 3D CINE MRI with self-gated Cartesian trajectory

**DOI:** 10.1016/j.mri.2016.12.021

**Published:** 2017-05

**Authors:** M. Usman, B. Ruijsink, M.S. Nazir, G. Cruz, C. Prieto

**Affiliations:** a King's College London, Division of Imaging Sciences and Biomedical Engineering, London, United Kingdom; b Pontificia Universidad Católica de Chile, Escuela de Ingeniería, Santiago, Chile; c Department of Computer Science, University College London, London, UK

**Keywords:** 3D cardiac CINE, Free breathing, Self-gating, Golden angle step

## Abstract

**Purpose:**

To present a method that uses a novel free-running self-gated acquisition to achieve isotropic resolution in whole heart 3D Cartesian cardiac CINE MRI.

**Material and methods:**

3D cardiac CINE MRI using navigator gating results in long acquisition times. Recently, several frameworks based on self-gated non-Cartesian trajectories have been proposed to accelerate this acquisition. However, non-Cartesian reconstructions are computationally expensive due to gridding, particularly in 3D. In this work, we propose a novel highly efficient self-gated Cartesian approach for 3D cardiac CINE MRI. Acquisition is performed using CArtesian trajectory with Spiral PRofile ordering and Tiny golden angle step for eddy current reduction (so called here CASPR-Tiger). Data is acquired continuously under free breathing (retrospective ECG gating, no preparation pulses interruption) for 4–5 min and 4D whole-heart volumes (3D + cardiac phases) with isotropic spatial resolution are reconstructed from all available data using a soft gating technique combined with temporal total variation (TV) constrained iterative SENSE reconstruction.

**Results:**

For data acquired on eight healthy subjects and three patients, the reconstructed images using the proposed method had good contrast and spatio-temporal variations, correctly recovering diastolic and systolic cardiac phases. Non-significant differences (*P* > 0.05) were observed in cardiac functional measurements obtained with proposed 3D approach and gold standard 2D multi-slice breath-hold acquisition.

**Conclusion:**

The proposed approach enables isotropic 3D whole heart Cartesian cardiac CINE MRI in 4 to 5 min free breathing acquisition.

## Highlights

• A novel self-gated 3D Cartesian acquisition is proposed for free breathing whole-heart cardiac MRI
• The proposed framework has efficient k-space sampling, better eddy current performance and high computational efficiency
• The Proposed method is able to achieve high spatio-temporal resolution 3D cardiac CINE
• The proposed method only requires four to five minute free breathing scan

## 1 Introduction

Multi-slice 2D CINE MRI is accepted as a gold standard for assessing cardiac function and anatomy. This approach requires multiple breath-holds and rigorous scan planning of multiple geometries (such as short axis and ventricular outflow tracts) and usually suffers from slice-misalignments due to a different breath hold position in the multiple acquisitions that may lead to erroneous assessment of ventricular volumes [1]. Moreover it has been shown that more than 30% of patients can have problems with holding their breath in a reliable and reproducible way [2]. A number of free breathing 2D CINE approaches [3], [4], [5], [6], [7] have been recently proposed that acquire data under free breathing and eliminate the need for breath-holds by correcting for any arbitrary respiratory motion in the reconstruction. By combining motion correction with accelerated imaging techniques, these approaches can achieve whole heart multi-slice coverage in 2 to 3 min free breathing acquisition. However, these multi-slice 2D CINE approaches have anisotropic spatial resolution and can only acquire data in a specific geometry. 3D CINE eliminates the need for CINE imaging in different planes (such as short-axis, two-chamber and four-chamber view and left and/or right ventricular outflow tracts), thereby reducing the overall planning and scan time. Due to isotropic resolution, 3D CINE allows reconstructed volumes to be reformatted into any plane for visualization. There are some techniques [8], [9] that try to acquire the whole 3D volume in a single breath-hold, but due to slow nature of MRI scanning, either spatial or temporal resolution is significantly reduced.

To overcome these problems, 3D free breathing navigator gated acquisitions have been proposed for cardiac imaging [10], [11], [12]. These approaches minimize respiratory motion by acquiring data within a small gating window at a pre-defined respiratory position (usually the end expiration). Respiratory gating prolongs scan time since only a fraction of the acquired data is accepted for reconstruction (referred to as scan efficiency) and requires a respiratory surrogate signal. Common respiratory surrogate signals include navigator echoes [12], [13], optical tracking [14] and respiratory bellows [10]. The navigator echo based approach performs well for free-breathing 3D coronary artery imaging where data are acquired in a short diastolic period of cardiac cycle. Optical tracking [14] and respiratory bellows [10] require long set-up times and careful calibration. For these reasons, self-navigating techniques [15], [16] are preferred in 3D cardiac CINE that can directly estimate the respiratory-induced cardiac motion from the acquired data itself.

Recently, several “free-running” 3D self-gated approaches have been proposed for different clinical applications including coronary, abdominal and CINE MRI. Examples of self-gated 3D non-Cartesian trajectories include 3D spiral phyllotaxis [17], [18], 3D Projection Reconstruction (PR) [19] and 3D stack of stars golden radial trajectory [20], [21]. For better signal contrast between blood and myocardium, especially required for coronary imaging, preparation pulses and fat saturation pulses are interspersed in the data acquisition [17], [18], [19]. For spiral phyllotaxis [17], [18] and 3D PR [19] trajectories, the respiratory self-navigation is usually obtained from a k-space readout along the superior inferior (SI) direction, regularly interleaved in the acquired data. 3D respiratory motion compensated images can be reconstructed with isotropic resolution that allows volumes to be reformatted into any plane for visualization. However, due to the 3D non-Cartesian sampling used, the computational complexity of reconstruction framework is much higher than Cartesian sampling based reconstruction. This can result in long reconstruction times particularly for non-linear reconstruction frameworks, including compressed sensing and total variation regularization [22], [23], where 3D gridding needs to be performed in each iteration of the reconstruction. For 3D stack of stars golden radial trajectory [20], [21], radial profiles are acquired at a given angle for all slices before moving to the next angle. A respiratory self-navigation signal can be obtained from 1D projection of centre k-space points of the radial profiles along the SI direction. This 1D respiratory signal can be used to produce respiratory gated images. However, this trajectory is also computationally demanding as multi-slice 2D gridding steps are needed in each iteration of the algorithm. Also, the trajectory is generally used in a specific short axis orientation with anisotropic resolution and hence does not allow for flexible visualization of any arbitrary user defined plane within heart.

Recently, 3D Cartesian trajectories including VDRad (Variable Density sampling and Radial view ordering) [24] and G-CASPR (Golden angle CArtesian acquisition with Spiral Profile ordering) [25] have been proposed for 3D abdominal MRI and coronary MRI, respectively. These trajectories acquire data along spiral-like interleaves on a Cartesian grid and have the advantage of low computational complexity. The golden angle (111.246°) between the consecutive interleaves ensures retrospective adjustment of temporal resolution by combination of any arbitrary number of profiles. In this work, we aim to achieve self-gated whole heart cardiac CINE MRI with a) Cartesian acquisition and b) isotropic resolution. Based on modification of G-CASPR trajectory, we propose a free-running self-gated 3D Cartesian acquisition called here as ‘CASPR-Tiger’ (CArtesian acquisition with Spiral PRofile ordering and Tiny golden angle step for eddy current reduction). Data is acquired continuously under free breathing (retrospective ECG gating, no preparation pulses interruption) using CASPR-Tiger trajectory. 4D volumes (3D + cardiac phases) are reconstructed from all acquired data (no respiratory data rejection) using a soft gating technique combined with temporal total variation (TV) constrained iterative SENSE reconstruction. Feasibility of proposed method is demonstrated in eight healthy subjects and three patients in a 4 to 5 min free breathing acquisition. Results are compared with multi-slice 2D breath-hold acquisition in terms of cardiac functional assessment.

## 2 Material and methods

Trajectories with golden angle step [26] are advantageous for free-running acquisitions as these allow retrospective adjustment of temporal resolution by combination of any arbitrary number of profiles. A golden-step 3D Cartesian acquisition with spiral profile order (G-CASPR) [25] has been recently proposed that samples the phase encoding plane following approximate spiral-like interleaves on a Cartesian grid. The order of sampling along each spiral-like interleave goes from the centre of k-space to the periphery and then to the centre k-space again for the next interleave. The angular step between two consecutive spiral interleaves is 111.246°, so-called golden angle. This trajectory enables reconstruction of high-resolution respiratory resolved images for application of coronary MR angiography, where data is acquired in mid-diastole for 100–120 ms with one spiral-like interleave per R-R interval. As data is acquired only in a mid-diastolic phase, the transient effects such as eddy currents originating from the golden step and periphery to centre k-space jump between the consecutive interleaves are expected to be diminished before the mid-diastolic data acquisition for the next interleave. However, for application of cardiac CINE where data is acquired continuously without interruption, in combination with balanced SSFP sequence, the golden angle step can lead to rapidly changing eddy currents, resulting in strong image artefacts [27]. Recently, a new sequence of smaller irrational angles (49.75°, 32.039°, 27.198°, 23.628°) called as *tiny golden angle* has been introduced for 2D golden radial acquisition [28]. Provided a sufficient number of k-space radial profiles are acquired, it has been shown that the smaller tiny golden angle of 23.628° results in k-space sampling distribution similar to standard golden angle of 111.246°, but with much better eddy current performance.

### 2.1 Proposed framework

**Fig. 1:**
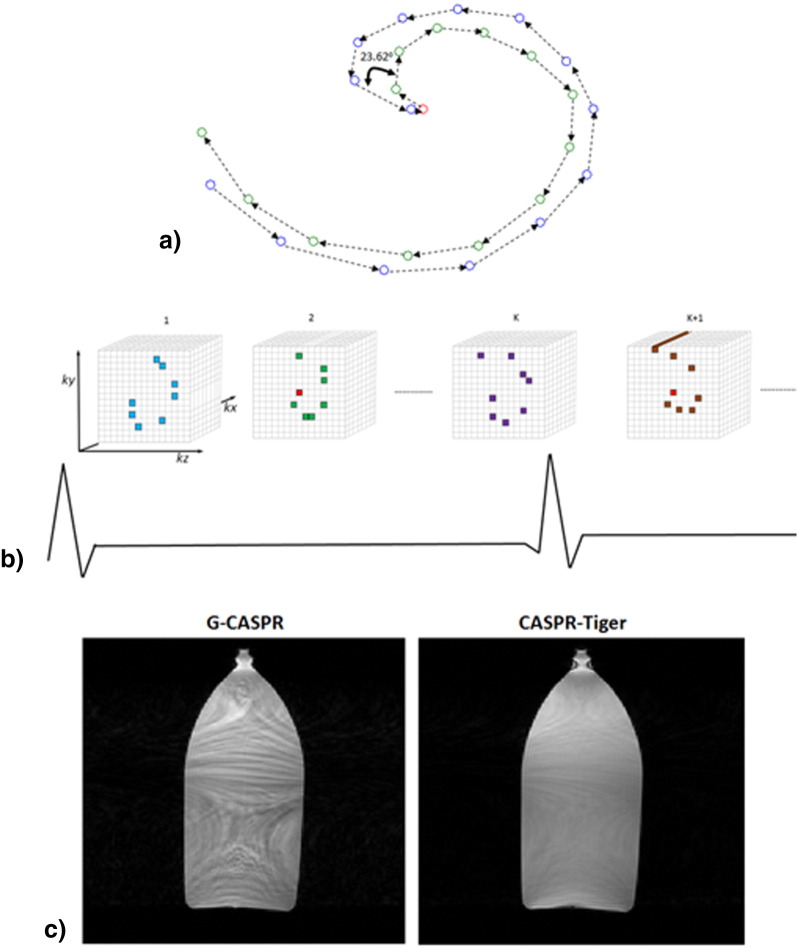
Free-running CASPR-Tiger acquisition: a) First interleave (blue) is acquired from outer to central k-space region, second interleave (green) is acquired in opposite direction. k-Space centre profile is acquired at each interleave pair (red). Each interleave is at 23.62° with respect to the previous one to ensure uniform k-space coverage and reduced eddy current artefacts. b) The acquisition is done continuously without any ECG triggering or respiratory navigator gating. c) Comparison of fully sampled reconstructions on phantom data acquired with free-running G-CASPR trajectory and proposed CASPR-Tiger acquisition, one slice selected from the 3D reconstruction is shown, most of the eddy current artefacts visible in G-CASPR reconstruction are diminished with the proposed approach.

Based on modification of G-CASPR trajectory, we propose a free-running self-gated 3D Cartesian trajectory called here ‘CASPR-Tiger’ (CArtesian acquisition with Spiral PRofile ordering and Tiny golden angle step for eddy current reduction). Instead of using the golden angle (111.246°), the trajectory acquires data continuously along spiral-like interleaves on a Cartesian grid, with tiny golden angle of 23.628° between the consecutive interleaves. To further avoid eddy current artefacts that can result from periphery to centre k-space jump between the consecutive interleaves, the interleaves are acquired in a paired fashion starting and finishing at the edges of k-space (Fig. 1a). The first interleave in each pair starts at the peripheral part of k-space and ends in the centre part of k-space. The second interleave starts in the centre k-space and ends in the outside part of k-space. For the purpose of self-respiratory navigation, the k-space centre is acquired at the beginning of second interleave in each pair (Fig. 1b). Fig. 1c shows a comparison of one slice selected from fully sampled 3D reconstructions from data acquired on a phantom with free-running G-CASPR trajectory and data acquired with proposed CASPR-Tiger trajectory. The eddy current artefacts that are visible in G-CASPR reconstructions are greatly reduced by the use of tiny golden angle in CASPR-Tiger trajectory. After the acquisition of a number of interleaves that populate full k-space (so called one full set), an arbitrary shift in the initial angle for each full set is introduced to minimize overlapped samples in the final reconstruction. The shift in the initial angle is computed as a fraction of tiny golden angle and is incremented in equal steps for each full set i as (i − 1 / N_full_set_) × 23.628°, where i = 1.2, …, N_full_set_; N_full_set_ denotes the total number of full sets. The respiratory signal is estimated from repeatedly acquired 1D projections and is used to define a reference bin at the end-expiration. The R-wave of the ECG is logged for the purpose of retrospective cardiac synchronization and data from different cardiac cycles are retrospectively combined using a linear model to reconstruct N different cardiac phases [29]. Soft-gating [24], [30] is performed to weight k-space data depending on respiratory displacement from the reference bin. Furthermore, the k-space data was motion-corrected in the SI direction by using 1D translational motion of the heart. The 1D translational motion of the heart in the SI direction was estimated using a template matching algorithm. A 1D region of interest (ROI) was manually defined that covered the heart along the SI direction. Template matching was performed between each ROI profile and a reference ROI profile by using normalized cross-correlation as the similarity measure with the first ROI profile being the reference. The resulting 1D signal for each coil is further filtered in the frequency range of 0.1–0.5 Hz to retain the respiratory component. The filtered signal in the coil element with the highest peak in the respiratory frequency range was selected as the 1D respiratory signal for motion correction. Using displacement values in the 1D signal, a 1D translational motion correction is performed by applying the corresponding phase-shifts in k-space, prior to reconstruction. Temporal total variation constrained iterative SENSE (TV-SENSE) [22], [23] reconstruction is done on the data with TV applied along the cardiac phase dimension. The reconstruction process can be formulated as:

(1) $${{\arg\min}_{x}\left\|W\left(\mathrm{Ex}-y\right)\right\|}_{2}^{2}+\lambda_{t}\;{\left\|\nabla_{t\;}x\right\|}_{1}$$

where **y** is the acquired data, **x** is the reconstructed 4D volume (3D + cardiac phases), **E** is the encoding operator that includes the coil sensitivities, Fourier transformation and sampling, ∇_**t** _ represents 1D temporal gradient, *λ*_*t*_ is a regularization parameter that is used to define balance between data consistency and TV regularization, and **W** performs soft gating by weighing each readout according to its respiratory displacement from the reference bin using a scaled Gaussian kernel with the maximum set to 1.

### 2.2 Experiments

The proposed acquisition was implemented on a 1.5 T scanner (Ingenia, Philips Healthcare). Whole-heart free breathing CINE acquisition was performed with the proposed approach in eight healthy subjects (age range: 21 to 35 years) and three patients (age range: 45 to 76 years) using a b-SSFP sequence. The acquisition was done on healthy subjects without the use of a contrast agent, whereas acquisitions on patients were performed after injection of gadolinium-based contrast agent (Gadovist, 0.1 mmol/kg). Written informed consent was obtained from all subjects according to institutional guidelines and the study was approved by the institutional review board. Relevant scan parameters include: flip angle = 50°, TR/TE = 3.5/1.7 ms, resolution = 2 mm isotropic, FOV = 350 × 350 × 90–120 mm^3^, number of coil elements = 28, number of spiral interleaves = 5000–7000 depending on the number of slices covering the heart, 14 readouts per spiral interleave, scan time = 4–5 min. In all scans, the SAR level was set to below 2 W/kg (the limit of the First Level Controlled Operating Mode according to IEC) at which medical supervision is not required. Coil sensitivity maps were estimated from a separate reference scan. A reference bin with width of 4 mm was defined at end expiration for soft-gating.

From the 1D motion corrected k-space data, sixteen cardiac phases were retrospectively reconstructed in all volunteers and patients using soft-gated TV-SENSE reconstruction. This resulted in temporal resolution ranging from 31 ms to 70 ms, depending on the heart rate of the subject. The overall acceleration factor for the free breathing scans was in the range from 3.5 to 4.0. The reconstructed 4D volumes were reformatted in different planes after reconstruction. As reference gold standard, multi-slice 2D fully sampled Cartesian breath-held data were acquired at the end-expiration in all the volunteers. Multi-slice 2D acquisition was ECG gated and performed in short-axis orientation. Relevant scan parameters include: flip angle = 50°, TR/TE = 3.5/1.8 ms, in-plane resolution = 2 × 2 mm^2^, slice thickness = 8 mm with no gap between slices, FOV = 350 × 350 × 90–120 mm^3^, cardiac phases = 16, number of coil elements = 28, scan time per breath-hold ~ 10 s.

For cardiac functional measurements, the reconstructed 4D volume with the proposed framework was reformatted into short axis plane. Left-ventricle (LV) functional measurements [31] including end-diastolic volume (EDV), end-systolic volume (ESV), ejection fraction (EF) and stroke volume (SV) were computed and compared with those obtained from the reference multi-slice 2D breath-hold images. The measurements were done by two clinicians trained in cardiac MR (with 5 years of experience) using manual segmentation of end-diastolic and end-systolic myocardial boundaries in each slice. Bland-Altman analysis [32] was used to assess the agreement between the measurements obtained with the proposed and multi-slice 2D methods in all healthy subjects. The differences of these measurements were tested with a two-tailed paired-sample *t*-test with a *P* value of less than 0.05 considered as statistically significant.

For healthy subjects, image quality of reconstructions with the proposed method and reference 2D BH gold-standard approach was qualitatively assessed based on the myocardial sharpness and residual artifact level. Two independent cardiologists trained in cardiac MR (with 5 years of experience) were asked to rank the sharpness of the boundary between the myocardium and blood pool on scale of 0 (extreme blurring) to 4 (no blurring). The residual artefact level in the reconstructed images was qualitatively assessed on the scale of 1 (worst) to 4 (best).

The TV-SENSE reconstruction was implemented in MATLAB (R2012b, The MathWorks, Inc., Natick, MA, USA) on a work station with a six core processor (Intel Xeon × 5670, 2.93 GHz, and 24 GB memory) using a nonlinear conjugate gradient (NLCG) reconstruction algorithm with backtracking line-search [33]. The optimal value of *λ*_*t*_ was determined empirically by comparing reconstructions with different *λ*_*t*_'s based on the balance between blurring artefacts and noise-like artefacts in the reconstructions.

## 3 Results

**Fig. 2:**
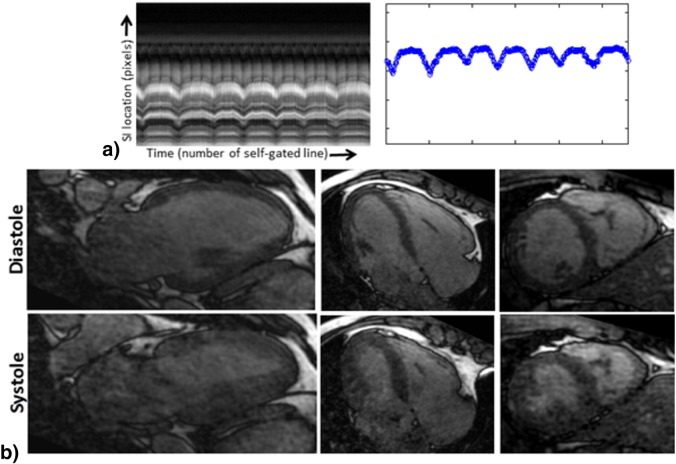
Self-gated 3D whole heart CINE MRI: a) 1D Superior-Inferior (SI) projection obtained from self-navigation in CASPR-Tiger trajectory and corresponding estimated 1D respiratory signal, b) from the reconstructed 4D volume, diastolic and systolic phases in two, four chamber and short axis orientations are shown for volunteer 1. Good quality reconstructions with isotropic resolution were observed in all orientations.

For one healthy subject, the 1D projection from centre k-space profiles and corresponding respiratory signal are shown in Fig. 2a. Reconstructed images for diastolic and systolic phases reformatted into 2-chamber, 4-chamber and short axis planes are shown in Fig. 2b. The proposed method corrected for most of the breathing artefacts, achieving good quality images in all plane orientations.

**Fig. 3:**
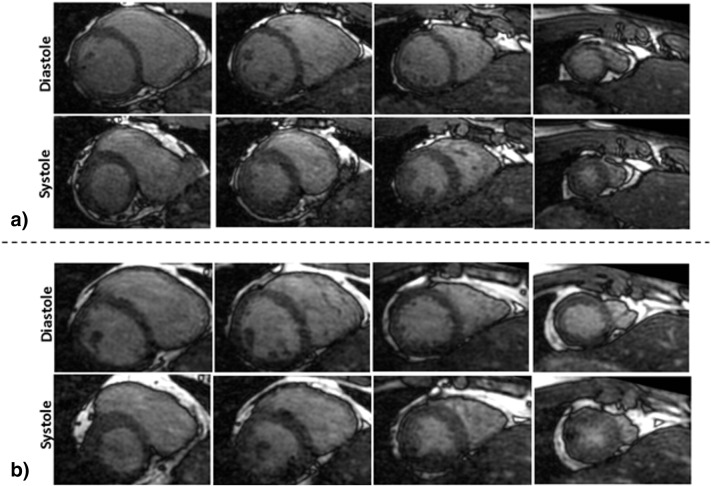
Results for two healthy subjects with proposed framework. Short axis slices from base towards apex in diastole and systole are shown for healthy subjects 2 and 3 in (a) and (b), respectively. The proposed 3D framework corrected for most of the respiratory motion artefacts in the reconstructed images, making them suitable for cardiac functional assessment.

**Fig. 4:**
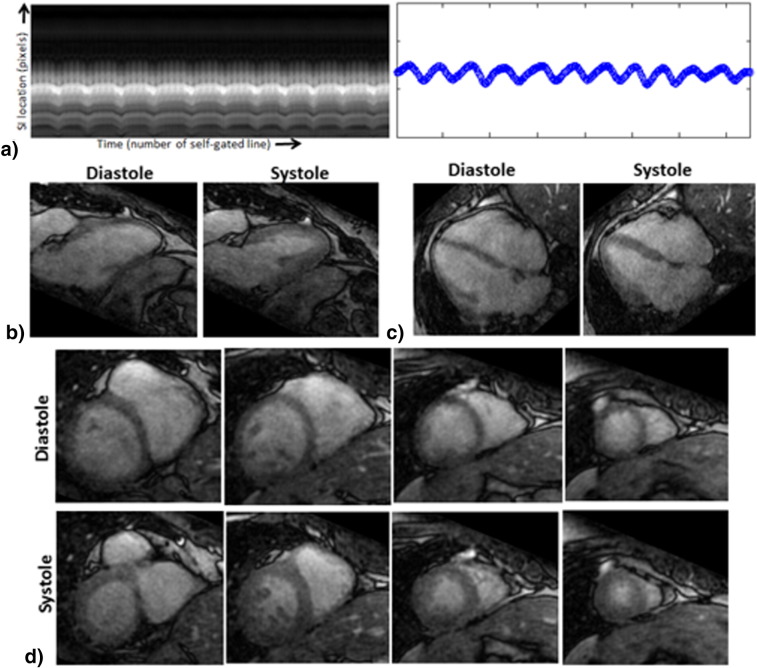
Results for a patient with proposed framework. a) 1D Superior-Inferior (SI) projection obtained from self-navigation in CASPR-Tiger trajectory and corresponding estimated 1D respiratory signal. From the reconstructed 4D volume, diastolic and systolic phases in b) two chamber, c) four chamber and d) short axis orientations are shown. Improved contrast was achieved between myocardium and blood pool in the reconstructed images in comparison to healthy subjects acquisitions.

For two healthy subjects, different slices in short axis orientation from 4D reconstructed volumes are shown in Fig. 3. The reconstructed images using the proposed method had good contrast and spatio-temporal variations from apical to basal slices, correctly recovering diastolic and systolic cardiac phases. Fig. 4 shows the 1D projection, respiratory signal and reconstructions in 2 chamber, 4 chamber and short axis planes for a patient. Due to the use of a contrast agent, the contrast in the reconstructed images was better in patients than in healthy subjects.

**Fig. 5:**
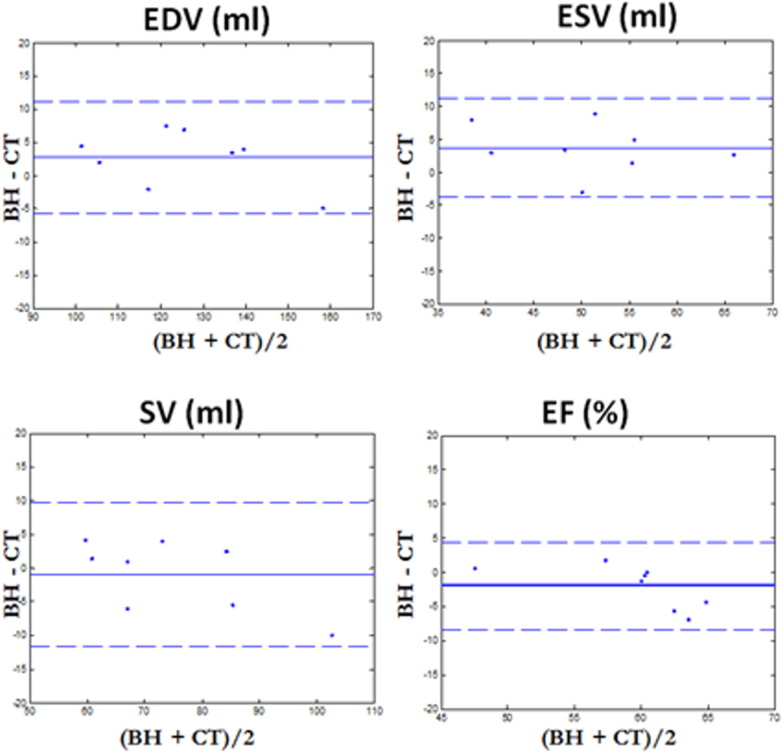
Bland-Altman plots for end-diastolic volume (EDV), end-systolic volume (ESV), stroke volume (SV) and ejection fraction (EF) for healthy subjects data. Reconstruction results with proposed framework are compared to reference multi-slice 2D breath-hold acquisition (BH). Along the plot axis, BH corresponds to reference breath-hold acquisition and CT corresponds to proposed framework. In each figure, mean value (middle solid line) and 2 standard deviation (top and bottom dashed lines) are shown. The proposed framework achieved similar quantitative cardiac functional values as for the BH reconstruction with no significant difference (*P* value > 0.05).

The mean and standard deviation of cardiac functional parameters measured from proposed method across eight healthy subjects are shown in Table 1. The LV functional parameters determined with the proposed method were in line with the values determined from the breath-hold reconstructions, with slight overestimation of EDV and ESV. The differences in all LV parameter values were not statistically significant (*P* > 0.05). The differences between the proposed and reference methods in terms of percentage error are also given. Bland-Altman plots for EDV, ESV, EF and SV measured from proposed method across eight healthy subjects are shown in Fig. 5. The Bland-Altman plots showed good agreement between the proposed 3D and reference 2D methods (EDV average difference: 2.7 mL, 95% confidence interval: [11.2:− 5.8] mL, ESV average difference: 3.7 mL, 95% confidence interval: [11.2:− 3.8] mL, SV average difference: − 1.0 mL, 95% confidence interval: [9.6:− 11.6] mL, EF average difference: − 1.9%, 95% confidence interval: [4.4:− 8.3] %). The values of LV functional parameters for all volunteers are listed in Table 2.

**Fig. 6:**
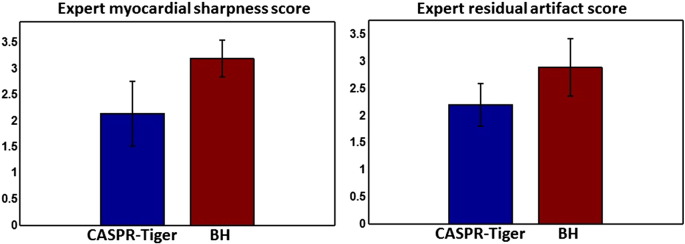
Image quality assessment of proposed 3D CASPR-Tiger method in healthy subjects: bar plots showing average expert scores for myocardial sharpness (0: extreme blurring to 4: no blurring) and residual artifact level (1: worst, 4: best) in the reconstructed images are shown. The associated standard deviations are also indicated. The results are compared with reference multi-slice 2D breath-hold (BH) reconstructions. Both myocardial sharpness and residual artifact scores were lower for proposed CASPR-Tiger method, when compared with reference BH method, but this did not have any major impact on cardiac functional parameters.

Bar plots comparing the qualitative average expert scores for the proposed CASPR-Tiger framework and reference BH techniques in terms of average myocardial sharpness and residual artifacts are shown in Fig. 6. Both myocardial sharpness and residual artifact scores were significantly lower for the proposed CASPR-Tiger method, when compared with reference BH method. However, this did not have any major impact on LV functional parameters as shown in Fig. 5 and Table 1.

## 4 Discussion

The proposed framework achieves whole heart 3D Cartesian CINE from four to five minute continuous acquisition under free breathing. Due to data acquisition with isotropic spatial resolution, the reconstructed volumes can be formatted into any user defined orientation for high resolution visualization. Compared to other recently proposed self-gating frameworks that use 3D non-Cartesian trajectories, the proposed technique does not interrupt the acquisition with preparation pulses and it is computationally more efficient as it uses a Cartesian based acquisition. With a non-optimized MATLAB based implementation, the average time for reconstruction of 4D volume from in-vivo free breathing data was 2.5 h. In comparison, a non-Cartesian trajectory based 3D free breathing CINE method [19] took around 16 h. The reconstruction times with non-Cartesian trajectory based frameworks are expected to be even worse (multiple days), if temporal regularization is also included in the reconstruction. In our TV-SENSE reconstructions, we found that the value of regularization parameter (*λ*_*t*_) in the range from 0.03 to 0.10 was adequate across all subjects giving a fair balance between blurring artefacts and noise-like artefacts in the reconstructions.

The proposed framework can be combined with channel compression techniques [34], [35] and Graphical Processing Unit (GPU) based implementation [36], [37] to reduce the reconstruction times to clinically acceptable range of 5 min. By using channel compression techniques [34], the size of parallel imaging data can be reduced, thereby reducing the reconstruction time without compromising the benefit of multiple coil elements. More specifically, a geometric decomposition coil compression (GCC) technique [35] has been recently proposed that minimizes the number of virtual coils (hence the reconstruction time) using a spatially varying coil compression. Coil compression is performed separately for each location along the fully sampled dimensions by a singular value decomposition (SVD). Then the spatially varying compression matrices are carefully aligned so that the virtual coils have smooth coil sensitivities. It has been shown that GCC based framework requires 14 times less computation than that of the original data, without image quality degradation. We expect our reconstruction times to be reduced from 2.5 h to approximately 10 min using GCC techniques. Further reduction in reconstruction times could be achieved by using GPU accelerated computers [36] that can execute algorithms in a massively parallel manner. It has been shown that compressed sensing reconstruction times can be shortened by a factor of 27 using highly parallelizable Split Bregman method combined with GPU computing platform [37]. By using a combination of channel compression and GPU implementation of proposed framework, the reconstruction of 4D whole-heart volume (3D + cardiac phases) could be possible to be achieved within less than a minute.

In multi-slice 2D breath-hold acquisitions, there is a time gap between subsequent acquisitions (every one or two slices) to allow for patient recovery and breathing instructions for the next scan. Taking this time into consideration, the overall duration for multi-slice 2D BH acquisition is in the range of 6 to 8 min. The acquisition with proposed 3D framework does not need to be performed under breath-hold and takes less amount of time than multi-slice 2D acquisition. Furthermore, there is no need for CINE imaging in different planes (such as short-axis, 2-chamber and 4-chamber view and left and/or right ventricular outflow tracts), hereby reducing the overall planning and scan time. As the 3D acquisition is undersampled by factor of 3.5 to 4.0, the reconstructed images with proposed framework have more residual artifact level (Fig. 6) when compared to the fully sampled multi-slice 2D reconstructions. However, this had no significant impact on cardiac functional measurements. The LV cardiac functional parameters obtained from the proposed 3D framework were in line with those obtained from the reference multi-slice 2D breath-hold acquisition with non-significant over estimation of EDV and ESV. One of the possible causes of this slight overestimation of LV EDV and LV ESV could be the use of temporal regularization term in TV-SENSE reconstruction that tends to smooth out the details and edges in the image if regularization parameters are not selected adequately. As we have done our studies in eight volunteers, further investigation is warranted in a larger group of study to establish the cause of slight non-significant overestimation.

With the proposed method, the combination of spiral-like interleaves results in uniform spatio-temporal pseudo-randomness needed for TV-SENSE reconstructions. However, in comparison with non-Cartesian trajectories such as 3D PR and spiral phyllotaxis, the incoherence of the sampling pattern is low resulting in some remaining noise-like artifacts. As compressed sensing reconstructions benefit more from variable-density random undersampling [30], [38], [39] than uniform random sampling, future works will focus on achieving better incoherence of sampling pattern by modification of CASPR-Tiger trajectory similar to VDRad trajectory [24] to acquire samples more densely in k-space centre than at the periphery.

In this framework, by using tiny golden angle, we have aimed at reducing eddy current artefacts originating from k-space jumps between consecutive spiral interleaves. The eddy current effects originating from k-space jumps within each interleave are not considered here, though they may not be negligible. Future works will focus on designing efficient trajectories for minimization of eddy current artefacts originating from k-space jumps both between and within the interleaves.

The proposed framework assumes 1D rigid motion of the heart along the SI direction and therefore uses simple 1D translational correction in k-space based on the displacement values of the self-gating signal. For soft-gating, although the framework uses all data for reconstruction, due to less corresponding weight W, the data at respiratory positions distant from end expiration will have little to no influence on the reconstruction. For better performance, a 3D motion compensation framework similar to the technique proposed in [3], [7] can be used that estimates 3D non rigid motion between different respiratory positions and performs motion corrected CINE reconstruction by integration of non-rigid motion directly in the reconstruction. Alternatively, a 5D motion resolved reconstruction [40] can be performed where images are reconstructed containing separated cardiac and respiratory dimensions. However, the motion compensation or motion resolved techniques remove motion artefacts at the expense of much increased computational complexity.

Due to the inflow of unsaturated blood in 3D CINE [41], [42], the contrast between myocardium and blood pool is inferior to that for the multi-slice 2D acquisition. Contrast between myocardium and blood pool can be increased by using contrast agents, as shown in our preliminary acquisitions in three patients. Future studies will be performed to validate the proposed method in clinical settings, where contrast agents are routinely used for cardiac MRI.

In our experiments, all our volunteers and patients had normal heart beat. In case of arrhythmia, based on the length of cardiac cycle, the ectopic cardiac cycles can be detected and the corresponding data should be excluded from the reconstruction. This will result in an increase in the net acceleration factor. Alternatively, for patients with high proportion of arrhythmic heart beats, data could be grouped for separate reconstructions from normal and ectopic cardiac cycle.

One of the limitations of the proposed framework compared to 3D PR or spiral phyllotaxis trajectory is that k-space profiles may be overlapping in combined k-space frames due to acquisition on a Cartesian grid. This means prolonged acquisition time as more data is needed to be acquired to satisfy TV-SENSE sampling requirement. In the proposed framework, this overlap is minimized by introducing a shift in the initial angle for each full set as a fraction of tiny golden angle. However, despite the overlap of samples being minimized in the combined k-space frames, it could not be totally avoided. In the future, we will investigate further to find an optimal shift to increase the sampling efficiency.

In our experiment, we have reconstructed 16 cardiac phases resulting in temporal resolution of 31 ms to 70 ms, depending on the heart rate of the subject. This is relatively a small number of cardiac phases compared to the current state of art multi-slice 2D techniques, where usually 25 to 30 cardiac phases are reconstructed. One solution could be to increase the number of reconstructed cardiac phases in the reconstruction and use view sharing approach as done in [8] with temporal width of multiple cardiac phases to remove aliasing artefacts. Further improvement of temporal resolution will be investigated in future works with optimized trajectories including variable density sampling and more sophisticated image reconstruction frameworks including respiratory motion compensated reconstruction [3], [7] and 5D motion resolved reconstruction [40].

## 5 Conclusion

In conclusion, a novel framework based on free-running 3D Cartesian self-gating trajectory is proposed that is suitable to achieve 3D cardiac CINE from four to five minute continuous free breathing acquisition. Feasibility of the proposed framework was demonstrated in eight healthy subjects and three patients.

## Figures and Tables

**Table 1:** Left ventricular functional parameters in eight healthy subjects. End-diastolic volume (EDV), end-systolic volume (ESV), stroke volume (SV) and ejection fraction (EF) for proposed 3D cardiac CINE framework and reference multi-slice 2D breath-hold reconstruction are given. The mean values of functional parameters are given together with the associated standard deviation. The *P* values quantifying the comparison of proposed method with reference fully sampled multi-slice breath hold reconstruction (BH) are also given. The differences between the proposed and reference methods in terms of percentage error are given. No significant differences were found between the values computed with the proposed method and those obtained from the breath-hold multi-slice 2D reference.

Results for healthy subjects				
Method	EDV (ml)	ESV (ml)	EF (%)	SV (ml)
CASPR-Tiger	126.9 ± 17.9	52.5 ± 8.5	58.5 ± 4.5	74.4 ± 13.1
BH	124.2 ± 19.7	48.8 ± 9.3	60.5 ± 6.5	75.4 ± 16.1
*P* value	0.7785	0.4201	0.4975	0.8951
Difference	3.76%	8.61%	6.07%	6.71%

**Table 2:** Left ventricular functional parameters in eight healthy subjects. End-diastolic volume (EDV), end-systolic volume (ESV), stroke volume (SV) and ejection fraction (EF) values for proposed 3D cardiac CINE framework (CASPR-Tiger) and reference multi-slice 2D breath-hold reconstruction (BH) are given.

Results for healthy subjects								
	EDV (ml)		ESV (ml)		EF (%)		SV (ml)	
Volunteer	CASPR-Tiger	BH	CASPR-Tiger	BH	CASPR-Tiger	BH	CASPR-Tiger	BH
1	141.50	137.50	56.00	54.50	60.42	60.36	85.50	83.00
2	125.00	117.50	50.00	46.50	60.00	60.42	75.00	71.00
3	103.50	99.00	42.00	39.00	59.42	60.60	61.50	60.00
4	106.50	104.50	42.50	34.50	60.09	66.98	64.00	70.00
5	129.00	122.00	67.30	64.50	47.83	47.13	61.70	57.50
6	116.00	118.00	48.50	51.50	58.19	56.36	67.50	66.50
7	155.65	160.50	58.00	53.00	62.73	66.97	97.65	107.50
8	138.50	135.00	55.90	47.00	59.64	65.18	82.60	88.00
